# Comparative Analysis between 3D-Printed Models Designed with Generic and Dental-Specific Software

**DOI:** 10.3390/dj11090216

**Published:** 2023-09-14

**Authors:** Cristian Abad-Coronel, Doménica Patricia Pazán, Lorena Hidalgo, Jaime Larriva Loyola

**Affiliations:** 1 CAD/CAM Materials and Digital Dentistry Research Group, Faculty of Dentistry, Universidad de Cuenca, Cuenca 010107, Ecuador; 2 Faculty of Dentistry, Universidad de Cuenca, Cuenca 010101, Ecuador; patricia.pazan16@ucuenca.edu.ec (D.P.P.); marial.hidalgo@ucuenca.edu.ec (L.H.); jaime.larrival@ucuenca.edu.ec (J.L.L.)

**Keywords:** CAD/CAM, dental software, 3D models, STL, digital workflow

## Abstract

With the great demand in the market for new dental software, the need has been seen to carry out a precision study for applications in digital dentistry, for which there is no comparative study, and there is a general ignorance regarding their applications. The purpose of this study was to investigate the accuracy differences between digital impressions obtained using generic G-CAD (general CAD) and D-CAD (CAD dental) software. Today, there is a difference between the design software used in dentistry and these in common use. Thus, it is necessary to make a comparison of precision software for specific and generic dental use. We hypothesized that there is no significant difference between the software for specific and general dental use. Methods: A typodont was digitized with an intraoral scanner and the models obtained were exported in STL format to four different softwares (Autodesk MeshMixer 3.5, Exocad Dental, Blender for dental, and InLAB). The STL files obtained by each software were materialized using a 3D printer. The printed models were scanned and exported in STL files, with which six pairs of groups were formed. The groups were compared using analysis software (3D Geomagic Control X) by superimposing them in the initial alignment order and using the best fit method. Results: There were no significant differences between the four analyzed software types; however, group 4, composed of the combination of D-CAD (Blender–InLAB), obtained the highest average (−0.0324 SD = 0.0456), with a higher accuracy compared to the group with the lowest average (group 5, composed of the combination of the Meshmixer and Blender models), a generic software and a specific software (0.1024 SD = 0.0819). Conclusion: Although no evidence of significant difference was found regarding the accuracy of 3D models produced by G-CAD and D-CAD, combinations of groups where specific dental design software was present showed higher accuracy (precision and trueness). The comparison of the 3D graphics obtained with the superimposition of the digital meshes of the printed models performed with the help of the analysis software using the best fit method, replicating the same five reference points for the six groups formed, evidenced a greater tolerance in the groups using D-CAD.

## 1. Introduction

The application of digital workflow in dentistry is increasing due to the rapid development and improvement of intraoral scanners, dental software and dental materials, becoming an integral part of the daily routine and communication between dentists and dental technicians [1,2]. In this field, one of the most well-known tools is CAD/CAM technology (computer aided design/computer aided manufacturing) [3,4], which offers among its advantages the ability to optimize processes that are more laborious analogically [5]. In addition, it makes it possible to design and manufacture chairside restorations, with high functional and aesthetic quality, ensuring a better fit between surfaces in a faster and more comfortable way for the patient, adjusting to the characteristics and anatomy of the tooth, by means of restorations designed with different softwares [6,7]. In summary, these technologies provide a more efficient workflow in the clinical environment, providing high accuracy, precision, predictability, efficiency and cost-effectiveness with a wide range of restorative materials with adequate physical, optical and biological properties [8,9]. The digital workflow with a CAD/CAM system involves image acquisition, digitization, design and manufacturing [10,11]. This workflow can be categorized into three groups: chairside, directly in the dental office; labside through a dental lab; or mixed, using processes from both of the above [12]. The process begins with the acquisition of information to create a “points cloud” by means of scanning. The spatial situation of the digitization of the scanned points is defined by their Cartesian coordinates; thus, a 3D model is formed, given by the union of the planes arranged in triangulations. This step is considered critical to originate a 3D file [13,14].

The points cloud generated during scanning is converted into a continuous surface through the CAD software algorithm, which may cause some loss of accuracy [12]. Technical factors influencing the accuracy of the scanning process include: ambient illumination, operating software version, the intraoral scanner’s optical impression technology and the depth of field and scanning strategy [3,15,16]. However, even if the point cloud has a low density or aberrant areas, the scanner can remove such measurements using computer algorithms, thus generating a better digital model [3,14,17]. For this reason, certain digitization software, after the acquisition of the image, is responsible for processing the information to generate a file that can be exported to different design programs depending on the format required [13,18]. The digital file obtained can be stored in various formats, such as stereolithography (STL), the 3D objects geometry information format (OBJ) and the polygon file format (TYP). OBJ or PLY files contain additional information concerning the color and texture of the object [19]. However, in most systems, CAD data are handled and transmitted in STL format, where each triangle of an STL file consists of three points with Cartesian coordinates, namely X, Y, Z and a surface [1], and has therefore become the standard file format in 3D printing [8]. In the CAM phase, the model can be materialized in various systems either as additive manufacturing (AM) in 3D printing or by subtraction through a milling machine [20].

In the exchange of data between different types of dental software there can be more or less accuracy in the mesh of the 3D objects [21]. The quality of the digital impression is defined by two independent factors, which are trueness or reliability and precision; the combination of both factors determines the accuracy [22]. Trueness is obtained by comparing the original geometry of the reference master model with the digitized model, while accuracy is obtained by an intra-group comparison of digitized models, i.e., it refers to the approximation of the agreement between test results [19,20]. During the acquisition and digitization steps, the accuracy of the impression may be affected [23]. 

There are two types of systems that support archives—open systems or closed systems. Closed systems offer a complete integrated flow, including data acquisition, virtual design with software and restoration fabrication in the same environment. All steps are integrated into a unique system, and there is no interchangeability between different systems from other companies [24], although some of them allow to export to software from different companies by exchanging STL files for design. Currently, many workflows allow opening universal files through the export of digital meshes in different formats [22,25].

On the other hand, open systems allow the adoption of the original digital data generated by CAD software and CAM devices from different companies, providing greater versatility [24,26]. These types of systems handle three-dimensional data in the STL format, which is the most commonly used in dental CAD/CAM systems, based on open source software because it is freely available, which means that any user can inspect, improve or share it. Its universal format allows STL to work with almost all CAD software programs [9,27].

When using open-source software, the fact that it may not have originally been developed for the use of dental design should be considered; this software has been transformed along time, as is the case of the software Blender 3.3.1, which was adapted to Blender for Dental 3.3.1 [28].

Software specifically for dental design can be classified as D-CAD and software for general non-specific design can be classified as G-CAD. The incorporation of new technologies in private practice or in a dental laboratory requires the mastery of design software, as well as the understanding of these tools and their application in various clinical situations and environments; it should be noted that the use of software involves a significant learning curve [29].

The CAD phase is a very challenging part of the digital workflow. The use of different types of specific dental design software added to the scarce existing literature has made it a subject to be investigated in depth [30]. The evaluation of D-CAD by analyzing the learning curve confirms that the results differ according to the type of software program [31]. New studies have shown that CAD learning is closely related to the learning curve and repeated D-CAD learning; therefore, software design learning is necessary for effective clinical application [32]. It should be considered that D-CAD learning is strongly influenced by the user interface (UI) of the software and the user experience (UX). Therefore, even if the software program is used for the first time, the better the UI and UX, the higher the learning rate [33].

D-CAD serves as an intuitive tool for professionals; however, the flexibility to create virtual designs is more limited due to the cost per package or version required, compared to the non-specific design software G-CAD. D-CAD has the advantages of presenting in a more simplified way all the tools available for designs; therefore, the learning curve and work time are shorter [34,35].

SGSD requires a longer learning curve and lower economic investment compared to D-CAD. However, the disadvantage of G-CAD is that it would be more prone to errors in both meshing and treatment planning [36]. In the absence of extensive literature on experimental studies of D-CAD, a dentist working with G-CAD software will encounter difficulties in comprehensive treatment planning, until he or she becomes familiar with all the necessary tools [37].

An example of G-CAD is MeshMixer, which is free with basic functions such as simple trimming, degumming, digital model labeling, and the option to add dental libraries; however, within its limitations, it cannot analyze occlusion, nor dental proportions, positions, shapes and morphologies. For this reason, the need for a longer learning curve is mentioned as a disadvantage, as well as a longer design time [27,29,38,39].

The purpose of this study was to compare the accuracy of different STL files of 3D models, which were exported with G-CAD and D-CAD, to observe if the meshes of the files could suffer modifications impacting the accuracy of the digital impressions. This would provide distinct information for dentists and laboratorians, besides providing criteria for their choice, in view of the scarce literature [27]. Given the different characteristics of the software used, the printed models were analyzed in terms of veracity and accuracy compared with the different types of design software. Therefore, the null hypothesis was that there would be no statistically significant differences in terms of accuracy and trueness between the files of the printed dental models that were designed with these two softwares.

## 2. Material and Methods

### 2.1. Model Digitalization and Design: First Phase

For the first phase, a master upper arch typodont was scanned with a high-end intraoral scanner (PrimeScan^TM^, Dentsply-Sirona^TM^, New York, NY, USA), following the scanning strategy recommended by the manufacturer. In addition, this method was considered due to the high trueness and the fact that the system seems to be a valid tool to obtain digital full-arch datasets in vivo with comparable accuracy [29]. It was then digitized, and the image obtained was exported in a high-resolution STL file format using a chairside software (CEREC 5.0.0, Dentsply-Sirona, NY, USA). 

The STL file was exported to each of the four different softwares previously selected: three SDEDs (Exocad Dental 3.1, exocad, Darmstadt, Germany; Blender for dental 3.3.1, blender, New York, USA; and InLAB SW 22.0, Dentsply-SironaTM, Bensheim, Germany); and one G-CAD (MeshMixer, Autodesk, San Francisco, CA, USA). Summarizing, the study compares the accuracy of the digital models processed with different CAD programs.

**Figure 1:**
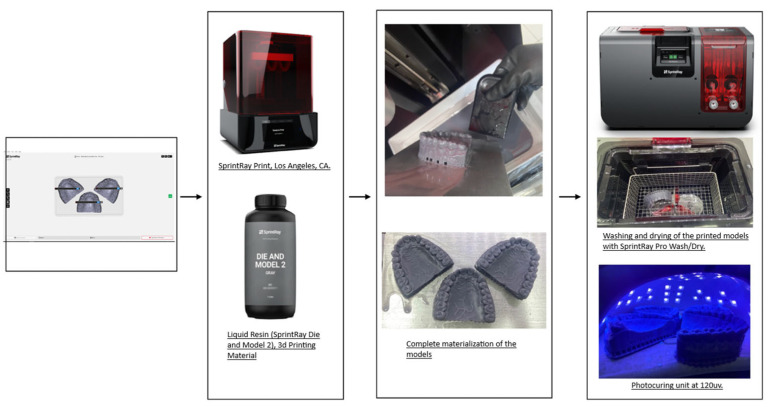
Model digitization and design procedure.

Next, an exported STL file was obtained from each design software studied. Each of the digital models were printed using a 3D printer (SprintRay, Los Angeles, CA, USA), with resin for models (SprintRay Die and Model 2). Four printed models for each group were obtained. Immediately, the printed models were placed on an automated multi-stage wash platform, starting with a two-cycle wash with the use of 91% isopropyl alcohol, followed by a 10-minute fast dry (SprintRay Pro Wash/Dry, SprintRay, Los Angeles, CA, USA); upon completion they were light-cured with 120 W UV light for 120 s in the built-in photo-polymerization system (Procure, SprintRay, Los Angeles, CA, USA) (Figure 1).

### 2.2. Digitization of the Model and Groups to Be Studied: Second Phase

The process began with the scanning of the four printed models, following the scanning strategy recommended by the manufacturer (PrimeScan^TM^, Dentsply-Sirona^TM^, New York, NY, USA). The digital models were exported as STL files in 100% resolution, in order to compare, by superimposing the meshes, the different softwares. To make the comparison, six groups were formed with the composition described in Table 1.

### 2.3. Analysis of .STL Files

Using mesh analysis software (Geomagic control; X Rock Hill/SC/3D Systems Inc., San Francisco, CA, USA), because it is a high-precision comparison software that generates various measurement points and color maps, it is easy for the designer to use. The two STL files formed in each group were imported for comparison, by initial alignment and the best fit method, replicating the same reference points. 

**Figure 2:**
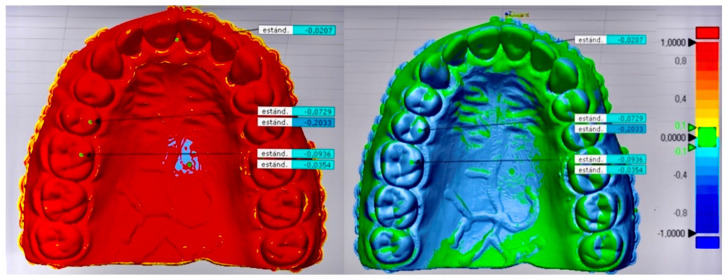
Group 6 (InLAB SW 22.0, Dentsply-Sirona, Bensheim, Germany and Autodesk MeshMixer, San Francisco, CA, USA).

From the aligned digital meshes, the 3D comparison was started, measuring five points in each group. The five measurement points were placed on the interincisal papilla, on the palatal cusp of pieces 1.5 and 2.5, and the mesiopalatal part of pieces 1.6 and 2.6. Replicating this step in the six groups for the same points, gave in total 30 measurement points, which provided the descriptive statistics of each group with mean, standard deviation, minimum and maximum values, in addition to the color map of −1 mm blue + 1 mm red, and the tolerance range of ±0.1 mm, as shown in Figure 2.

### 2.4. Statistical Analysis

For data analysis, the variables obtained from the combinations of the six groups of software used in this study were defined. Based on the data generated by the analysis software, statistical tests of homoscedasticity (equality of variances) were performed with Levene’s statistical test, a normality test was performed with the Shapiro–Wilk test, and a test of ranges was performed with the Kruskal–Wallis statistical test (to determine discrepancies in the average), all with statistical software (SPSS V27 for Windows; IBM Corp. Chicago, IL, USA) and with a significance level of 5%.

## 3. Results

Table 2 shows the descriptive statistics, where group 4 obtained the highest average (−0.0324 SD = 0.0456) with minimum and maximum values of −0.070 and 0.0462, respectively. From these results it was observed that groups 3 and 4 showed greater precision and accuracy with less dispersion between their measurements.

**Figure 3:**
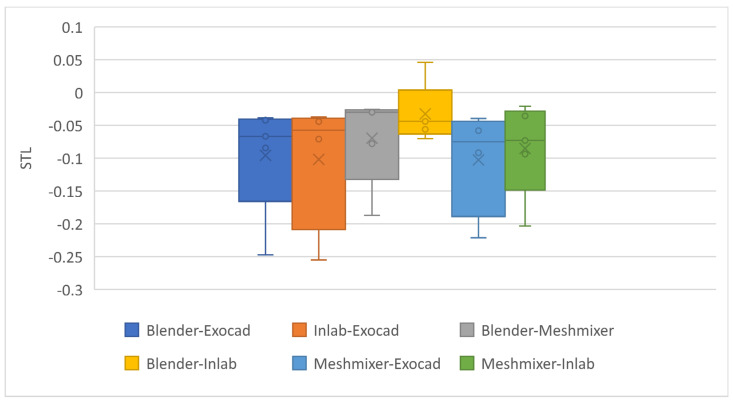
Box plot for STL measurements of the groups. G1: Blender–Exocad; G2: InLAB–Exocad; G3: Blender–Meshmixer; G4: Blender–InLAB; G5: Meshmixer–Exocad; G6: Meshmixer–InLAB.

Figure 3 shows the analysis of outliers and quartiles. In this case, no outliers were found in any of the groups. For group 1 (Blender–Exocad) little dispersion was observed; the median showed that 50% of the measurements were below −0.0665, quartile 1 indicated that 25% were below −0.1656 and quartile 3 showed that 75% were below −0.0403. Group 2 (InLAB–Exocad) was the group with the largest dispersion of all, where 50% of the observations were below −0.0574, 25% below −0.2088 and 75% below −0.0389. Group 3 (Blender–Meshmixer) was the group with the second-lowest dispersion, where 50% of the observations were below −0.0299, 25% below −0.1325 and 75% below −0.0260. Group 4 (Blender–InLAB) was the group with the lowest dispersion of all (highest accuracy), where 50% of the observations were below −0.0439, 25% below −0.063 and 75% below 0.004. Group 5 (Meshmixer–Exocad) was the second group with the highest dispersion after group 2, where 50% of the observations were below −0.0747, 25% below −0.1886 and 75% below −0.044. For group six, 50% of the observations were below −0.0729, 25% below −0.1485 and 75% below −0.0281. According to these results, the values were similar between groups, but some groups were more variable than others. The results of the descriptive analysis indicated that the Blender for Dental, InLAB, MeshMixer, and Exocad softwares showed greater accuracy and precision in the group combinations where a D-CAD was present. However, the null hypothesis was not rejected, meaning there is no evidence of a significant difference in the accuracy of 3D dental models.

Table 3 shows the normality test of the measurements. According to these values, normal distribution was confirmed for group 4 (*p*-value = 0.079 > 0.05), group 5 (*p*-value = 0.210 > 0.05) and group 6 (*p*-value = 0.314 > 0.05). Therefore, the nonparametric Kruskal–Wallis test was used to determine the mean discrepancy.

The Kruskal–Wallis test for independent samples did not reject the null hypothesis. With respect to the standard deviation, the Levene test did not reject the null hypothesis of equality of variances, that is, there were no significant differences between the standard deviations of the groups. One of the objectives was to determine whether there were significant differences between the average obtained with each group. Table 4 shows that there were no differences between the average of each group (H = 3.524 and *p*-value = 0.620 > 0.05). Therefore, the null hypothesis was accepted.

Table 5 showed that there were no differences between the precisions (standard deviation) reported by the different groups, since the Levene statistic based on the mean and median yielded values greater than the significance level (mean *p*-value = 0.772 and median *p*-value = 0.977).

**Figure 4:**
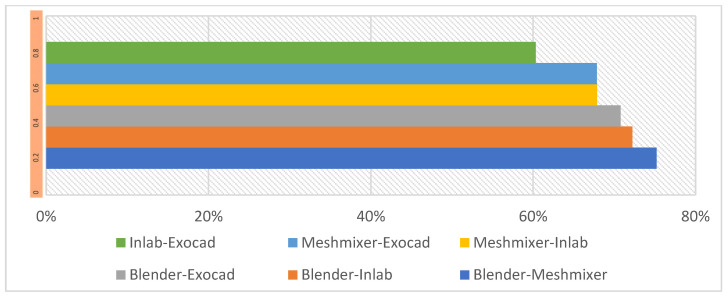
3D tolerance bar diagram. G1: Blender–Exocad; G2: InLAB–Exocad; G3: Blender–Meshmixer; G4: Blender–InLAB; G5: Meshmixer–Exocad; G6: Meshmixer–InLAB.

From the analysis of Figure 3 and Figure 4, it was determined that group 3 showed the highest percentage within the tolerance range of 75.21%, followed by group 4 (72.23%) and group 1 (70.78%). Groups 6, 5 and 2 showed tolerances of 67.87%, 67.85% and 60.32%, respectively, showing lower tolerance and greater dispersion.

## 4. Discussion

The purpose of this study was to compare both D-CAD and G-CAD softwares through the accuracy of different STL files of 3D models, to observe the behavior of the meshes of the files that could influence the veracity of the digital impressions. 

The results of the descriptive analysis indicate that the Blender for dental, InLAB, MeshMixer and Exocad softwares showed higher accuracy and precision in the combinations of groups in which a D-CAD was present. However, the null hypothesis was not rejected, meaning that there is no evidence of significant difference in the accuracy of dental 3D models produced by different softwares, both D-CAD and G-CAD.

Regarding the average, the D-CAD groups obtained higher accuracy and lower dispersion than the average obtained with a G-CAD. 

By standard deviation, the study also found no significant differences between the groups. These findings suggest that the choice of dental software used to fabricate dental 3D models may not be critical in terms of precision and accuracy, as all the softwares evaluated produced comparable results. However, the findings through outlier and quartile analysis of the STL measurements for each group did not provide outliers for any of them, as similar results were identified, even though some groups showed more variability than others. Therefore, the results provided by the different softwares for both the D-CAD or G-CAD softwares included in the study did not show significant differences, although better results in terms of precision and accuracy were evidenced when compared to a D-CAD.

The comparison of the 3D graphics obtained with the superimposition of the digital meshes of the printed models performed with the help of the analysis software using the best fit method, replicating the same five reference points for the six groups formed, evidenced a greater tolerance in the groups using a D-CAD.

Although the use of digital impressions in dentistry is not a new topic, there remains a concern about dispensing with conventional impressions and relying more on digital impressions. Several studies have compared the accuracy of digital impressions of dental implants with conventional impression techniques and the results obtained have shown that the accuracy offered by digital impressions can be clinically acceptable [40,41]. In this regard, they noted that results can be influenced by the operator (e.g., experience and scanning strategy) [21,42,43], by technology (e.g., scanners, printers, algorithms, software) [44,45], and clinical conditions (e.g., ambient light, dental materials within the oral cavity, saliva and/or blood, amount of attached gingiva, patient movement) [46,47].

Specifically in relation to the technologies involved as part of the CAD/CAM process, there is evidence from previous studies investigating the accuracy of the different scanners on the market [48,49,50], which revealed that the results of the accuracy of digital impressions with intraoral scanners may vary depending on whether the investigation was performed in vitro [51] or in vivo [52]; few studies addressed the accuracy achieved by combining the scanner with CAD design software, in the cases of those with proprietary or open source design software considering the deviations that occur when exporting the STL file to different CAD design software that make it possible to evaluate data loss during the transfer of intraoral scans to various CAD design programs. According to this approach, one study concluded that more accurate results are obtained when using the proprietary design software associated with the intraoral scanner used, and in another case they recommend the use of open system scanners that perform a direct export to STL format because data loss related to model accuracy was observed in their study when transferring from the proprietary scanner format to the STL format [53,54]. The effect of scanner software versions and CAD design software on the pre-accuracy of the results has also been examined, showing that the most recent updates guarantee greater accuracy and therefore more satisfactory results. Regarding the accuracy of STL files, software updates seem to achieve increasingly accurate STL files [55,56].

Accuracy refers to precision and trueness. Precision, conceptually, is the difference between repeated measurements on a given target, while trueness expresses how close the results of a measurement are to the real values of the measured object [57]. In relation to fingerprints, trueness is an important measure for analyzing a model from this source [27,58]. This is while the evidence of accuracy of digital impressions according to the literature consulted would be clinically acceptable between 50 and 120 μm [59,60,61], which means that the accuracy of the digital impression as a first step in any digital workflow should be below that range. It is noteworthy that the accuracy of today’s digital impressions has led to their integration into dental offices [7]. Accuracy can be measured by different methods, with many cases evaluating accuracy by examining prosthetic workflows [59,62]. However, most of the research similar to the present study used special software to compare STL files with a reference data set [57], with best-fit alignment being the most commonly used method. In this sense, it is revealed that the checking software (Geomagic, Control X) used in this study has shown good accuracy in the measurement of digital models [58,63,64].

Technological progress in recent years has led to the implementation of a large number of D-CAD and G-CAD softwares, and the intraoral scanner used in the study has a good reputation in relation to the accuracy of the measurements. In the case of G-CAD, the new developments have been adapted and now include extensive dental libraries, as is the case of MeshMixer, which facilitates its use in dental design. In general, the software that makes dental design possible currently offers different tools, protocols for recording CT or CBCT data, surface models and outputs in STL format, and makes it possible to integrate 2D design into 3D, as is the case of Exocad Dental, InLAB and Blender for dental, applications that have the capacity to make dental pieces with complex geometry, making maximum use of resources [65] and carrying out virtual planning using the entire digital workflow.

According to other authors, some software designs can be more intuitive than others, making their choice very subjective, recommending that before choosing a system, as many software designs as possible should be tested until finding the most satisfactory CAD program, which best fits the specific systems used in the daily routine [66,67], where the user considers for their choice costs, the tools offered, the learning curve, the time consumption for the design and its affinity with the digital flow [8,39,68]. These reasons supported the interest of the present research in view of the scarce literature comparing generic and specific software [69].

It should be noted that in the present in vitro study, the scanning was performed in an environment different from the intraoral conditions; therefore, the results obtained may differ if performed under clinical conditions. A limitation of the study is also identified as the use of a single intraoral scanner and 3D printer, which limits the results of the study to the technical specifications of the software and hardware of this equipment. However, it is important to highlight that the intraoral scanner used in the study has a good reputation in relation to the accuracy of the measurements. In relation to this, the Roth study revealed that, in the comparison between 12 intraoral scanners, CEREC Primescan in relation to the accuracy results (trueness + precision) was the most accurate (4.2 trueness points + 3.2 precision points = 7.4 points out of 10) [70,71], which presents results comparable with the accuracy of laboratory scanners mainly in short and linear segments [72]. Regarding the importance of the printer used to establish a comparison, Morón et al. stated that statistically significant differences can occur in the accuracy of the printed models, with better results for industrial desktop 3D printers than for dental ones [73]. However, printers such as the DLP printer used in this study have increasingly automated material selection and subsequent post-production processes with integrated devices.

**Figure 5:**
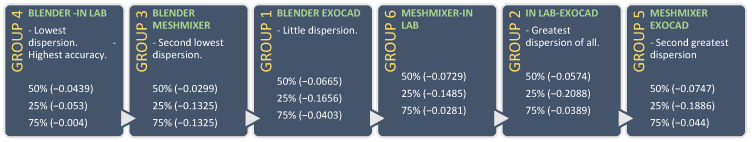
Dispersion between groups. G4: Blender–InLAB; G3: Blender–Meshmixer; G1: Blender–Exocad; G6: Meshmixer–InLAB; G2: InLAB–Exocad; G5: Meshmixer–Exocad.

Regarding the results obtained for the different softwares, other authors, in findings concordant with our results, showed that the three CAD programs analyzed in their study (InLAB, Multi-CAD and Blue-Sky CAD) can design clinically acceptable crowns in terms of internal and marginal fit, although the InLAB crowns outperformed the others in terms of marginal fit [74], showing that statistically there were no significant differences between the results obtained by the different design softwares; however, InLAB, which is a D-CAD, produced the most accurate results (Figure 5).

In terms of tolerance, the D-CAD softwares showed better results. This is in agreement with other authors’ results, in addition to highlighting their versatility, high precision and greater operator intuitiveness. 

Among the limitations of the study, in addition to being an in vitro study and therefore lacking intraorally reproducible clinical conditions (saliva and darkness, among other factors), a single operator, a single scanner model and a single 3D printer were used. As a recommendation, it is necessary to increase the sample size, to have greater certainty in the results.

Therefore, further studies are required to analyze and compare the results of different design softwares used in dentistry.

Although the study provides valuable information on the accuracy of various dental softwares, further research with a higher sample size is necessary to confirm these results and assess other relevant aspects such as user-friendliness, efficiency, and cost. Ultimately, the choice of dental software should be based on a variety of factors, such as the user’s needs, the complexity of the case, and resource availability, and not solely on the accuracy and precision of the produced 3D models.

## 5. Conclusions

The present study has shown that the softwares evaluated in this work presented similar results in terms of dispersion measures, as well as uniformity in the color maps generated by groups from the different combinations of STL files. In addition, it was shown that there were no alterations in the performance of the different softwares used in the study, suggesting that there are no significant differences in accuracy and veracity between the files of the printed models that were designed with three specific and one general softwares; however, it should be noted that the groups that were combined with a D-CAD had better accuracy than the G-CAD.

▪ The results of the descriptive analysis indicated that the softwares Blender for dental, InLAB, MeshMixer and Exocad showed greater accuracy and precision in the combinations of groups in which a D-CAD was present.
▪ The comparison of the 3D graphs obtained with the overlap of digital meshes, using the analysis software with the best fit method, showed greater tolerance in the groups that used the D-CAD and showed better results.
▪ Regarding the mean, the D-CAD groups obtained greater precision and lower dispersion than the mean obtained with the G-CAD.

## Figures and Tables

**Table 1:** Groups included in the study.

Software Paired Groups		
Group 1	Exocad Dental, Darmstadt, Germany.	Blender for dental, New York, NY, USA.
Group 2	InLAB SW, Dentsply-Sirona, Bensheim, Germany.	Exocad Dental, Darmstadt, Germany.
Group 3	Autodesk Mesh Mixer, San Francisco, CA, USA.	Exocad Dental, Darmstadt, Germany.
Group 4	Blender for dental, New York, NY, USA	InLAB SW, Dentsply-Sirona, Bensheim, Germany.
Group 5	Autodesk Mesh Mixer, San Francisco, CA, USA.	Blender for dental, New York, NY, USA
Group 6	InLAB SW, Dentsply-Sirona, Bensheim, Germany.	Autodesk Mesh Mixer, San Francisco, CA, USA.

**Table 2:** Descriptive statistics of the STL for the six groups. G1: Blender–Exocad; G2: InLAB–Exocad; G3: Blender–Meshmixer; G4: Blender–InLAB; G5: Meshmixer–Exocad; G6: Meshmixer–InLAB.

Statistics	Groups					
	G1	G2	G3	G4	G5	G6
Mean	−0.0956	−0.1017	−0.0694	−0.0324	−0.1024	−0.0852
Standard deviation	0.0866	0.1031	0.0693	0.0456	0.0819	0.0721
Variance	0.0075	0.0106	0.0048	0.0021	0.0067	0.0052
Minimum	−0.2470	−0.2548	−0.1870	−0.0700	−0.2210	−0.2033
Maximum	−0.0384	−0.0372	−0.0254	0.0462	−0.0393	−0.0207

**Table 3:** Normality test for STL values in the groups.

Cluster	Statistical	*p*-Value
Blender–Exocad	0.737	0.023 < 0.05
InLAB–Exocad	0.744	0.034 < 0.05
Blender–Meshmixer	0.746	0.027 < 0.05
Blender–InLAB	0.799	0.079 > 0.05
Meshmixer–Exocad	0.845	0.210 > 0.05
Meshmixer–InLAB	0.881	0.314 > 0.05

**Table 4:** Kruskal–Wallis test for STL values in the groups.

Cluster	Average Range	Kruskal–Wallis H	*p*-Value
Blender–Exocad	12.20	3524	0.620 > 0.05
InLAB–Exocad	12.50		
Blender–Meshmixer	17.80		
Blender–InLAB	18.60		
Meshmixer–Exocad	10.75		
Meshmixer–InLAB	2.00		

**Table 5:** Levene’s test for homoscedasticity of STL values across groups.

	Levene’s Statistic	Gl1	Gl2	*p*-Value
Based on the average	0.502	5	22	0.772 > 0.05
Based on the median	0.152	5	22	0.977 > 0.05

## Data Availability

https://drive.google.com/drive/folders/1Tul0ifjtGoEDQ1k7z8dqZwsGlD369vOB?usp=share_link.
